# Linkage to care following a home-based HIV counselling and testing intervention in rural South Africa

**DOI:** 10.7448/IAS.18.1.19843

**Published:** 2015-06-08

**Authors:** Reshma Naik, Tanya Doherty, Debra Jackson, Hanani Tabana, Sonja Swanevelder, Donald M Thea, Frank G Feeley, Matthew P Fox

**Affiliations:** 1 Health Systems Research Unit, South African Medical Research Council, Tygerberg, South Africa; 2 Department of Global Health, School of Public Health, Boston University Boston, MA, USA; 3 Population Reference Bureau, Washington, DC, USA; 4 School of Public Health, University of the Western Cape, Bellville, South Africa; 5 Department of Public Health Sciences, Karolinska Institutet, Stockholm, Sweden; 6 Division of Community Health, Faculty of Health Sciences, Stellenbosch University, Stellenbosch, South Africa; 7 Biostatistics Unit, South African Medical Research Council, Tygerberg, South Africa; 8 Center for Global Health & Development, Boston University Boston, MA, USA; 9 Department of Epidemiology, Boston University Boston, MA, USA

**Keywords:** HIV/AIDS, cascade of care, continuum of care, linkage to care, treatment, CD4, home-based, HIV testing, South Africa, test and treat

## Abstract

**Introduction:**

Efforts to increase awareness of HIV status have led to growing interest in community-based models of HIV testing. Maximizing the benefits of such programmes requires timely linkage to care and treatment. Thus, an understanding of linkage and its potential barriers is imperative for scale-up.

**Methods:**

This study was conducted in rural South Africa. HIV-positive clients (*n*=492) identified through home-based HIV counselling and testing (HBHCT) were followed up to assess linkage to care, defined as obtaining a CD4 count. Among 359 eligible clients, we calculated the proportion that linked to care within three months. For 226 clients with available data, we calculated the median CD4. To determine factors associated with the rate of linkage, Cox regression was performed on a subsample of 196 clients with additional data on socio-demographic factors and personal characteristics.

**Results:**

We found that 62.1% (95% CI: 55.7 to 68.5%) of clients from the primary sample (*n*=359) linked to care within three months of HBHCT. Among those who linked, the median CD4 count was 341 cells/mm^3^ (interquartile range [IQR] 224 to 542 cells/mm^3^). In the subsample of 196 clients, factors predictive of *increased* linkage included the following: believing that drugs/supplies were available at the health facility (adjusted hazard ratio [aHR] 1.78; 95% CI: 1.07 to 2.96); experiencing three or more depression symptoms (aHR 2.09; 95% CI: 1.24 to 3.53); being a caregiver for four or more people (aHR 1.93; 95% CI: 1.07 to 3.47); and knowing someone who died of HIV/AIDS (aHR 1.68; 95% CI: 1.13 to 2.49). Factors predictive of *decreased* linkage included the following: younger age – 15 to 24 years (aHR 0.50; 95% CI: 0.28 to 0.91); living with two or more adults (aHR 0.52; 95% CI: 0.35 to 0.77); not believing or being unsure about the test results (aHR 0.48; 95% CI: 0.30 to 0.77); difficulty finding time to seek health care (aHR 0.40; 95% CI: 0.24 to 0.67); believing that antiretroviral treatment can make you sick (aHR 0.56; 95% CI: 0.35 to 0.89); and drinking alcohol (aHR 0.52; 95% CI: 0.34 to 0.80).

**Conclusions:**

The findings highlight barriers to linkage following an increasingly popular model of HIV testing. Further, they draw attention to ways in which practical interventions and health education strategies could be used to improve linkage to care.

## Introduction

Linkage to care following HIV diagnosis is a critical step in the continuum of HIV care [1–4]. Timely linkage to care and treatment by HIV-positive individuals can lead to decreases in morbidity and mortality, as well as increases in life expectancy and quality of life [5,6]. Further, there are important prevention benefits as early initiation on antiretroviral treatment (ART) can significantly reduce HIV transmission to uninfected partners [7,8]. Modelling exercises suggest that universal HIV testing coupled with immediate treatment could decrease HIV incidence and virtually eliminate the HIV/AIDS pandemic [9].

To achieve these benefits, the rate of linkage to care must be very high [9], yet a systematic review of linkage to care in sub-Saharan African HIV programmes shows that only 59% (range 35 to 88%) of clients who test HIV positive complete the first step in the process. Even fewer remain in care until they initiate HIV treatment [4]. This fact underscores the importance of understanding and addressing barriers to timely linkage. Although high dropout rates at each stage of the HIV care continuum are common [4], little is known about the reasons for this.

Current evidence suggests that home-based HIV counselling and testing (HBHCT) is an economically sensible [10,11], acceptable and effective [10,12–21] strategy for raising awareness of HIV status. It is also increasingly recognized as a viable platform for implementing the “test and treat” strategy [22]. Thus, the mandate for scale-up is strong, and the next step is to ensure that appropriate measures are put in place to maximize the benefits of timely linkage after HBHCT. However, there is a dearth of information on linkage to care following HBHCT to inform such measures.

To address this gap in the literature, we set out to determine what proportion of clients who tested HIV positive in an HBHCT programme in rural South Africa linked to care within three months and which socio-demographic factors and personal characteristics influence the rate of linkage to care following HBHCT.

## Methods

### Setting and intervention

This study was conducted between September 2009 and January 2011 in the Umzimkhulu municipality – a poor, rural area in Kwazulu-Natal, South Africa. It was part of a larger cluster randomized controlled trial of door-to-door HBHCT called “Good Start,” which has been described in detail elsewhere [12,23]. In 19 intervention clusters (eight of which were part of the Good Start trial), trained lay counsellors offered free rapid HIV testing to all consenting household members aged 18 years or older and to those aged 14 to 17 years with parental or guardian consent. About 9.7% (*n=*492) of all clients who received HIV testing at home tested HIV positive.

**Figure 1:**
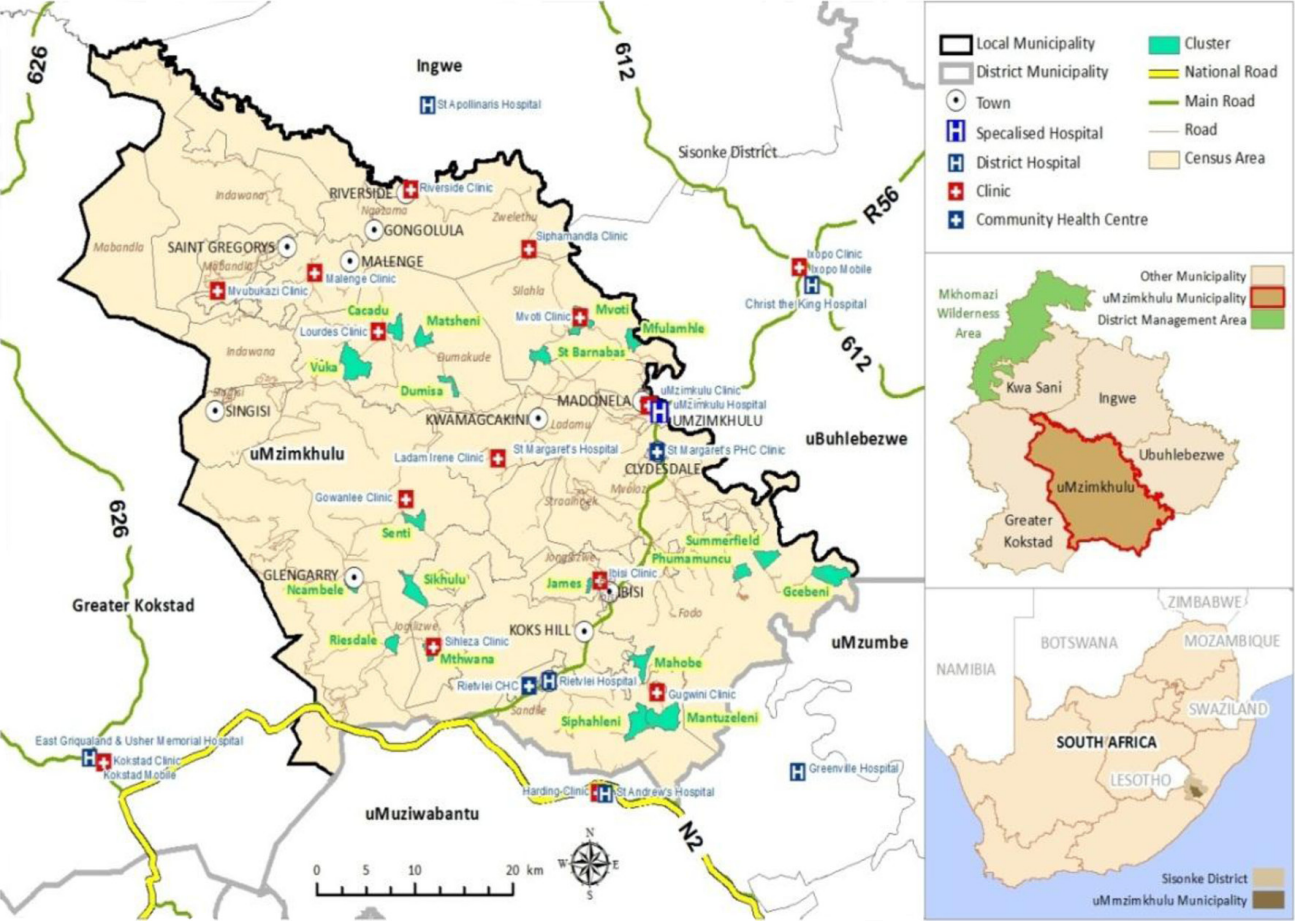
Map showing HBHCT intervention clusters and nearby health facilities.

For the current linkage to care study, the sampling frame included all 492 clients who tested HIV positive in the 19 HBHCT intervention clusters. Clients who tested HIV positive were offered post-test counselling, briefed on the importance of obtaining a CD4 count and given a referral letter to take to a health facility of their choice to undergo CD4 testing. The catchment area for this study population is served by nine primary healthcare clinics, one district hospital and one specialized TB hospital (Figure 1). At the time of the study, all health facilities drew blood for CD4 counts and samples were sent off-site for laboratory testing. HIV-positive clients were followed up over time to monitor linkage to facility-based HIV care.

### Study design and variables

This study included two primary analyses: (1) a prospective cohort analysis of linkage to care within three months and (2) a Cox regression analysis to determine factors associated with the rate of linkage to care. For both analyses, we defined the outcome of linkage to care as visiting a health facility and giving a blood sample for a CD4 count. Outcome data was collected in two different ways: (1) during periodic home visits or phone calls, counsellors used a paper-based monitoring tool to record self-reported follow-up information and (2) the study nurse tracked referral letters and followed up on self-reported clinic attendance by checking and obtaining information from official registers at the health facilities. Extensive efforts were put into follow-up including at least three attempted contacts for each client, frequent visits to clinics and thorough cross-checking of facility records. For those who linked to care, verified clinic data was used for the final outcome when available and self-reported data was used for the rest. For those who reported not linking to care and whose referral letter was not found at a clinic, the self-report was used as the final outcome.

For the Cox regression, data on predictor variables was collected from two sources. The first was a participant survey conducted by the lay counsellors at the time of testing. Variables taken from this survey included number of adult household members, gender, age, marital status and whether an HIV test had been taken prior to HBHCT. The second data source was a cross-sectional survey implemented midway through the Good Start study with 196 HIV-positive clients, 6 to 153 days following HIV testing (median: 16 days). This survey was designed using a socio-ecological framework based on a literature review of factors that could potentially influence linkage. The broad themes explored included demographic characteristics, life circumstances (i.e. number of people living in the household or for whom client is a caregiver), experience of home-based testing, barriers to accessing health care, perceptions about the healthcare system, knowledge and beliefs about HIV/AIDS and ART, fear and stigma, social support and disclosure, mental health (i.e. depression symptoms) and alcohol use.

### Statistical analysis

For the prospective cohort analysis, we used a binary outcome (yes or no) of linkage to care within three months of HBHCT. To determine the proportion of HIV-positive clients with this outcome, we first excluded all clients who were completely lost to follow-up or lost to follow-up prior to three months, or who were found to have been in pre-ART or ART care prior to home-based HIV testing. Note that we did include clients who reported having tested HIV positive previously but not having linked to care. To determine the disease stage of clients who did link to care, we calculated the median CD4 count (cells/mm^3^) at the initial point of entry into care. We also calculated the proportion of clients who linked to care (within any time period) who were eligible for treatment based on guidelines in South Africa during the time of the study (threshold set at ≤200 cells/mm^3^) [24] and based on current guidelines (threshold set at ≤350 cells/mm^3^) [25].

For the Cox regression analysis, the event of interest was linkage to care as defined previously. Person-time for each client who linked to care was calculated as time (in days) from HBHCT to the date of the CD4 count and, for those who did not seek care, as time from HBHCT to the last available date of follow-up. For 23 clients with a missing CD4 date, the time to CD4 was estimated as the midpoint between the date of testing and the first date the counsellor learned that the CD4 had been obtained. Twenty clients who were not included in the prospective cohort analysis because they were lost to follow-up prior to three months were included in the survival analysis because they did have some person-time to contribute.

Cox regression was used to determine which candidate predictor variables were significantly associated with linkage to care. Models were adjusted for clustering of data within communities using shared frailty models. To generate our final regression model, we included variables that had values of *p*≤0.1 in bivariate analysis. A backward elimination stepwise procedure was then carried out to produce a final model, which retained all variables significantly associated with linkage to care (*p*<0.05).

To ensure that the proportional hazards assumption was not violated, tests of proportional hazards were carried out both graphically and by using goodness-of-fit tests for each variable in the final model. Cox-Snell residual testing was carried out to ascertain that no outlying observations were disproportionately affecting the outcome.

### Ethical approval and consent procedures

Ethical approval for the study was received from the Institutional Review Board of the Boston University Medical Campus and the Ethics Committee of the South African Medical Research Council. Consent procedures varied for different components of the study. For HIV testing, clients provided written informed consent or a thumbprint, a procedure conforming to the district's protocol at the time. For collection of basic demographic and test result data, clients were read an information sheet and gave verbal consent. For participation in the cross-sectional survey, clients were read and given a written information sheet in the local dialect and provided written consent or a thumbprint. For review of medical records at health facilities, clients provided written informed consent or a thumbprint at the time of testing.

## Results

**Figure 2:**
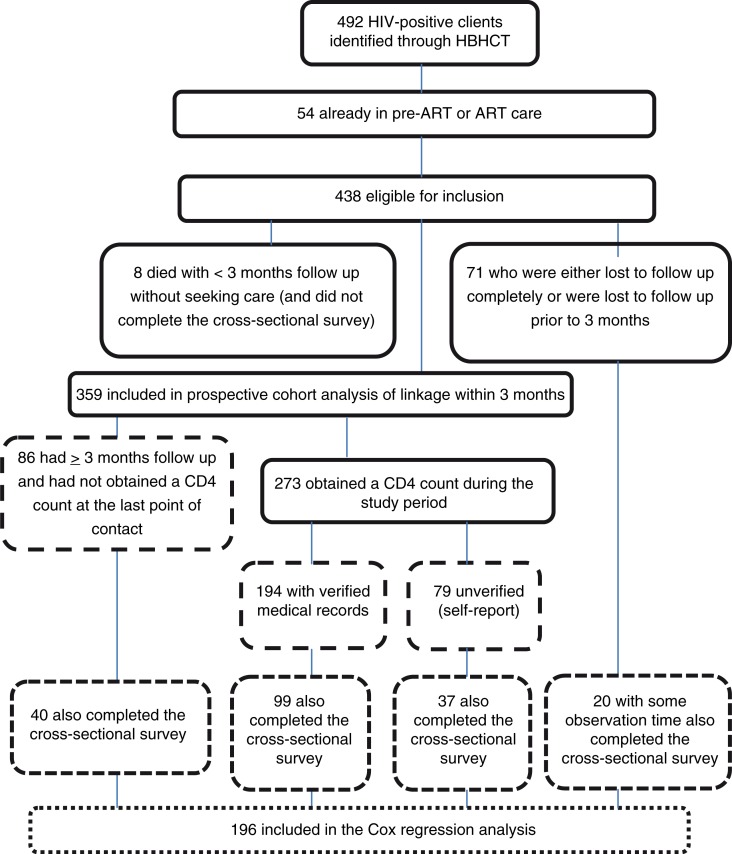
Study profile.

Figure 2 shows the study profile, which describes inclusions and exclusions in each of the two primary analyses. Of the initial 492 clients, 54 (11%) were found to already be in pre-ART or ART care prior to testing, leaving 438 subjects eligible for inclusion. Among the 438 eligible, we excluded 8 who died (none had sought care before death and all had less than three months of observation time) and 71 who were completely lost to follow-up or who were lost to follow-up prior to three months. Most of the clients lost to follow-up had moved from the study area, were unreachable by phone or refused follow-up. After exclusions, 359 clients remained for the prospective cohort analysis. This number included 22 clients who had tested HIV positive prior to HBHCT but who had not linked to care.

The background characteristics of the full sample of HIV-positive HBHCT clients and for the subsamples used for each of the two main analyses are shown in Table 1. The comparison illustrates that clients included in each analysis are generally representative of the full sample. Notably, the majority were female (83%), of reproductive age and from small households. The sample included a relatively equal number of single and married people, and just below 50% had not previously tested for HIV.

**Table 1:** Comparison of characteristics for full sample of HIV-positive HBHCT clients and those included in each stage of analysis

Characteristic	All HIV-positive HBHCT clients (*N=*492) *n* (%)	Clients included in the prospective cohort analysis (*N=*359) *n* (%)	Clients included in the Cox regression analysis (*N=*196) *n* (%)
Number of adults living with client			
<2	305 (62.0)	227 (63.2)	123 (62.8)
≥2	187 (38.0)	132 (36.8)	73 (37.2)
Gender			
Male	101 (20.5)	63 (17.6)	40 (20.4)
Female	391 (79.5)	296 (82.4)	156 (79.6)
Age group			
15 to 24	110 (22.4)	73 (20.3)	30 (15.3)
25 to 49	303 (61.6)	224 (62.4)	123 (62.8)
50+	79 (16.1)	62 (17.3)	43 (21.9)
Marital status			
Single	220 (44.7)	156 (43.4)	73 (37.2)
Married/co-habitating	202 (41.1)	146 (40.7)	89 (45.4)
Divorced/separated/widowed	70 (14.2)	57 (15.9)	34 (17.4)
Ever tested prior to HBHCT			
Yes	265 (53.9)	181 (50.4)	88 (44.9)
No	227 (46.1)	178 (49.6)	108 (55.1)

HBHCT: home-based HIV counselling and testing.

### Prospective cohort analysis

Of the 359 eligible clients, 273 or 76.0% (95% CI: 71.6 to 80.4%) had a final outcome of linkage to care, meaning they obtained a CD4 count at some point during the study period (i.e. between 0 and 542 days after HBHCT). Verified clinic records were found for 194/273 (71.1%) of these clients. For our primary outcome of interest, 223/359 or 62.1% (95% CI: 55.7 to 68.5%) of clients linked to care within three months. Of note, 17/22 or 77% (95% CI: 59.4 to 94.6%) of the clients who knew their HIV-positive status prior to HBHCT linked to care within three months.

In order to confirm robustness of the mixed self-report and clinic-verified data, a sensitivity analysis was conducted by limiting the sample to 280 clients (194 with a clinic-verified date of CD4 count and 86 who did not link to care). This analysis produced very similar results. In that case, 60.4% (95% CI: 54.7% to 66.1%) obtained a CD4 count within three months.

Of the 273 clients who linked to care, most (82.8%, 226/273) had a recorded CD4 count. Among those 226 clients, the median (interquartile range [IQR]) CD4 count was 341 cells/mm^3^ (IQR 224 to 542 cells/mm^3^). Findings were essentially unchanged when limited to only those 194 clients with a clinic-verified CD4-count (median 340 cells/mm^3^). Of the clients with a CD4 count, 45/226 (20%) were eligible for treatment using the eligibility threshold of ≤200 cells/mm^3^ that was used during the study period. According to the current eligibility threshold of ≤350 cells/mm^3^, 119/226 (53%) would be eligible.

### Cox regression analysis

**Figure 3:**
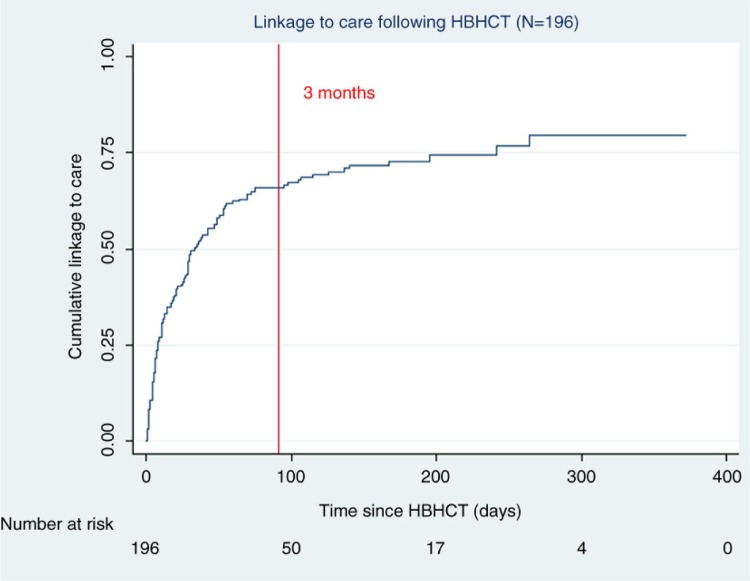
Cumulative linkage to care curve.

Of the 492 clients included in the prospective cohort, 218 had tested prior to the start of data collection for the Cox regression, leaving 274 eligible for inclusion. After excluding those already in pre-ART or ART care (*n=*18) and those completely lost to follow-up (*n=*51), there were 205 clients remaining. Of those, 196 also had one or more days of observation time and had completed the cross-sectional survey. These clients were included in the Cox regression analysis (Figure 2). Of the 196 clients, 69.4% (*n=*136) linked to care (i.e. obtained a CD4 count) and 30.6% (*n=*60) did not. The median duration of observation time for *all* clients was 29 (IQR 8 to 24) days, and for those who did not link to care it was 139 (IQR 48 to 191) days. Figure 3 shows the overall cumulative linkage-to-care curve. Most linkage occurred early, within the first three months after testing positive, with little additional linkage after three months.

In the bivariate Cox regression analysis, we found several factors that were associated with the rate of linkage. Through multivariable analysis (Table 2), we found that believing that drugs or supplies are generally available at the local health facility (adjusted hazard ratio [aHR] 1.78; 95% CI: 1.07 to 2.96); experiencing three or more depression symptoms post diagnosis (aHR 2.09; 95% CI: 1.24 to 3.53); being a caregiver to four or more people (aHR 1.93; 95% CI: 1.07 to 3.47) and knowing someone who died of HIV/AIDS (aHR 1.68; 95% CI: 1.13 to 2.49) were all predictive of *increased* incidence of linkage to care.

**Table 2:** Multivariate associations of client characteristics and linkage to care among HIV-positive HBHCT clients (*N=*196)

Variable	*n* (%)	Number of clients who linked to care/person-days	Rate of linkage per 100 person-days	Unadjusted HR (95% CI)	Adjusted HR (95% CI)
Number of adults living with client					
<2	123 (62.8)	90/6562	1.4	1	1
≥2	73 (37.2)	46/6364	0.7	0.62 (0.43 to 0.89)	0.52 (0.35 to 0.77)
Age group					
15 to 24	30 (15.3)	13/3081	0.4	0.41 (0.23 to 0.73)	0.50 (0.28 to 0.91)
25+	166 (84.7)	123/9845	1.3	1	1
Number of people for whom client is a caregiver					
0 to 3	27 (13.8)	13/2540	0.5	1	1
4+	169 (86.2)	123/10385	1.2	1.85 (1.04 to 3.27)	1.93 (1.07 to 3.47)
Believed HBHCT results?					
Yes	141 (71.9)	112/7611	1.5	1	1
No/not sure	55 (28.1)	24/5315	0.5	0.37 (0.24 to 0.58)	0.48 (0.30 to 0.77)
Finding time to go to the facility					
Not a problem	155 (79.1)	114/9211	1.2	1	1
Big/small problem	41 (20.9)	22/3715	0.6	0.56 (0.35 to 0.88)	0.40 (0.24 to 0.67)
Perceptions drug/supply availability					
Never/rarely/sometimes	40 (20.4)	21/4310	0.5	1	1
Mostly/always	104 (53.1)	80/5627	1.4	2.05 (1.26 to 3.33)	1.78 (1.07 to 2.96)
Unknown/never been to clinic	52 (26.5)	35/2989	1.1	1.85 (1.07 to 3.20)	1.83 (1.03 to 3.28)
ART can make you feel sick					
Agree	40 (20.4)	25/3811	0.7	0.65 (0.42 to 1.02)	0.56 (0.35 to 0.89)
Disagree/unknown	156 (79.6)	111/9115	1.2	1	1
Know someone who died of HIV/AIDS?					
Yes	124 (63.3)	93/7817	1.2	1.42 (0.98 to 2.04)	1.68 (1.13 to 2.49)
No	72 (36.7)	43/5109	0.8	1	1
Number of mental health symptoms after HBHCT					
1 to 2	35 (17.9)	18/3001	0.6	1	1
3+	161 (82.1)	118/9925	1.2	1.69 (1.03 to 2.77)	2.09 (1.24 to 3.53)
Drink alcohol?					
Yes	56 (28.6)	33/3918	0.8	0.69 (0.47 to 1.03)	0.52 (0.34 to 0.80)
No	140 (71.4)	103/9008	1.1	1	1

HBHCT: home-based HIV counselling and testing; HR: hazard ratio; ART: antiretroviral treatment.

We also found that younger age (15 to 24 vs. 25+ years) (aHR 0.50; 95% CI: 0.28 to 0.91); living with two or more adults (aHR 0.52; 95% CI: 0.35 to 0.77); not believing or being unsure about the HIV test results (aHR 0.48; 95% CI: 0.30 to 0.77); difficulty finding time to seek health care (aHR 0.40; 95% CI: 0.24 to 0.67); believing ART can make you sick (aHR0.56; 95% CI: 0.35 to 0.89); and drinking alcohol (aHR 0.52; 95% CI: 0.34 to 0.80) were all predictive of *decreased* incidence of linkage to care.

## Discussion

For test and treat strategies to be effective at reducing HIV incidence, novel approaches such as HBHCT will be important for identifying HIV-positive clients. However, if rates of linkage to care are low, as has been seen with those who test in mobile or stand-alone facilities [4], the benefits may be far less than expected. This study, one of the few to report on linkage to care following home-based HIV testing, showed that approximately 62.1% (95% CI: 55.7 to 68.5%) of HIV-positive HBHCT clients obtained a CD4 count within three months following testing. This finding is similar to studies in resource-rich settings [26], as well as resource-poor settings where other models of testing were used [4,27]. A recent systematic review, including 10 studies conducted in sub-Saharan Africa, found that the median proportion of clients linking to care within two to three months was 59% (range: 35 to 88%). Our finding is well within that range and suggests that although linkage after HBHCT appears no worse than in patient-initiated testing programmes in other settings, there is still more work to be done.

Currently, there is limited data on linkage to care following HBHCT. Among the extant studies, rates of successful linkage range from 42 to 96% [27–29]. Our rate of linkage is in the middle of this range, but it is difficult to make direct comparisons as each study used a different definition of linkage. Two of the three previous studies that achieved higher rates of linkage used point-of-care CD4 count testing at the time of HIV testing and defined linkage as seeking the next step in care. It could be that having a clear sense of their current health status motivated clients to seek further care or treatment, whereas in our study linkage meant taking the first step to obtain the CD4 count armed only with the knowledge of HIV status. In addition to differences in the outcomes measured, our study was also conducted in an area with numerous study communities and referral clinics, variable and difficult geographic terrain and high population mobility. This factor may also explain at least some of the difference in rates of linkage.

Our study focused on the transition from HIV testing to CD4 staging. Because this is only the first of several phases where clients could be lost before receiving needed care and treatment, our rate of linkage is far from optimal. However, given that home-based testing generally achieves higher testing coverage than facility-based testing, achieving similar rates of linkage could result in a net gain in the number of people entering treatment. Linkage rates in the upper range of those found within facility-based settings is also very encouraging considering the poor, rural infrastructure of our setting, the inherent challenges to linkage from the home to a health facility and the likelihood that many clients would not have tested in the absence of the home-based intervention – and thus may have been less prepared for a positive result.

We identified several personal and health system factors that are both positively and negatively predictive of linkage to care. Some of these factors are amenable to intervention, whereas others provide important insights about subgroups that may need particular attention. One area that is amenable to intervention is client responses to the HIV test results. For example, we found that clients who did not believe the results of their HIV test were less likely to link to care. In addition, although the number was small, the majority of those clients who knew their HIV-positive status prior to HBHCT linked to care within three months, thus it could be that having more time to process and come to terms with the results was an influential factor. Together, these findings suggest that counsellors within HBHCT programmes should place particular emphasis on assessing clients’ responses to their results and finding ways to guide them to acceptance. Interventions to improve knowledge about treatment may also be effective given that clients who believed that ART could make you sick were less likely to link to care.

Another area amenable to intervention is around time and opportunity costs. We found that clients who reported that finding time to seek health care was either a “big” or “small” problem were less likely to link to care than those who reported it was not a problem. At the same time, clients who lived with two or more adults were less likely to link to care than those living alone or just with one adult. It could be that those living with more adults have greater household responsibilities and also less opportunity to leave the home for long periods without explanation. The latter could pose a particular challenge if they have not disclosed their status to all household members. These findings suggest that interventions designed to facilitate disclosure, reduce stigma and ease the time and logistic burdens of care-seeking may improve retention.

In regard to more general views on health or the healthcare system, we found that clients who believed that drugs and supplies were available at their local health facility or even those with no knowledge about drug and supply availability were more likely to link to care than those who believed that they were not available. This finding is not surprising considering that poor perceptions of the healthcare system appear to hinder healthcare utilization more generally in many parts of sub-Saharan Africa [30–34]. This factor underscores the importance of overall health system improvement.

This study also draws attention to particular subgroups that may need extra attention to ensure linkage to care. For example, similar to other studies [35,36], we found that adolescents had a much lower incidence of linkage to care than older adults. Youth may face unique emotional and logistic challenges to care-seeking that require tailored interventions. For example, they may struggle more with acceptance of their status or disclosure and lack the social support or financial resources to seek care. Clients who drink alcohol may also have unique life circumstances that hinder linkage and that counsellors may need to better understand and address. Those who drink may be less likely to seek care because of impaired judgment, or drinking itself may be a sign of poor self-care. Current evidence shows that alcohol use is associated with poor adherence to ART [37,38] and enhanced disease progression [39] and may also influence the use of drugs to treat common opportunistic infections associated with HIV [40].

This study has important strengths. This is one of only a few studies to date investigating linkage to care following HBHCT. Second, the study was conducted in a “real world” setting with a relatively poor rural population, broad geographic area with weak transport infrastructure, numerous referral clinics and clinic activities operating as per usual. Thus, the findings offer a good indication of what can be achieved in a typical rural African context.

Despite its strengths, the findings from this study must be interpreted in light of some important limitations. First, the inclusion of unverified data may have led to an overestimate in the proportion of clients who linked to care if not all clients were being truthful. However, we did have verified records for 71.1% of clients who linked to care, which is a considerable proportion. Further, the decision to include self-reports took into account important contextual factors including the following: clients sometimes sought care at clinics outside of our catchment areas; referral letters were found at the clinic for some clients and clinic records in this area were often incomplete. In addition, because self-reporting of date of CD4 and actual CD4 results may be subject to recall bias, the proportion of clients seeking care within three months and the median CD4 could be mismeasured.

Another important consideration is that selection bias due to the exclusion of clients who moved, refused follow-up or who were otherwise lost to follow-up prior to three months may have affected the analysis of timely linkage. Such clients may have different care-seeking behaviour and in particular may be at risk of delayed or non-linkage to care due to stigma or other social factors. However, we did conduct several sensitivity analyses (not shown) that included the 71 clients lost to follow-up. Even when we assumed that as few as 25 (35%) linked to care within three months, the new 95% confidence interval still includes our original point estimate of 62.1%, indicating that it is relatively robust.

Importantly, because women comprised a particularly large proportion of study participants (due to labour migration patterns among males, which led to a high preponderance of women participating in the larger HBHCT study [12]), it limits our ability to generalize the findings to men. We also recognize that disclosure is likely to be an influential factor with regard to care-seeking but were not able to include it in the multivariable analysis due to variability in methods and timing in measuring it. We were, however, able to document that by the end of the study, at least 244/359 (68%) of clients had disclosed their status.

A further limitation of this study is that we are unable to determine temporality of the associations because, in some cases, the cross-sectional survey may have taken place after linkage occurred. Finally, the sample size used for the Cox regression analysis was relatively small and thus may have lacked power to detect all of the relevant relationships between the predictor variables and outcome. The small sample size also prohibited stratification by gender and may have been subject to selection bias similar to the primary analysis.

## Conclusions

A drastic increase in HIV testing and treatment rates is needed for the achievement of national and international goals to combat HIV/AIDS. Given the growing body of evidence about the acceptability and effectiveness of community-based HIV testing models such as HBHCT, the likelihood of scale-up throughout sub-Saharan Africa is high. In order to maximize the benefits of such interventions, timely linkage to care is critical. Overall, our findings are encouraging, showing that HBHCT can identify a substantial number of previously undiagnosed HIV-positive clients and that over half are eligible for treatment soon after testing. Despite the challenging setting, rates of linkage to care were comparable and importantly no worse than those found within facility-based settings. Given that home-based testing is known to increase testing coverage rates, this could result in considerable net gains in linkage. Finally, our findings highlight important gaps in our understanding about factors that can influence linkage. They suggest that certain subgroups such as youth may require particular attention and also draw attention to areas where practical interventions and strategies could be implemented to address clients’ needs and improve linkage to care following community-based HIV testing.
